# Asthma Exacerbations: Integrative Analysis of miRNA Activity Using Single-Cell Transcriptomics

**DOI:** 10.64898/2026.07.31.741637

**Published:** 2026-08-06

**Authors:** Parham Hadikhani, Xiting Yan, Geoffrey Chupp, Ga Young Ban, Shraddha Piparia, Michael McGeachie, Rinku Sharma, Scott T. Weiss, Louise C. Laurent, Alvin T Kho, Kelan G. Tantisira

## Abstract

**Background:**

Asthma exacerbations are caused by dysregulated cellular interactions between airway and immune cell populations. Circulating microRNAs (miRNAs) are potential biomarkers for asthma exacerbations; however, their target airway cells remain poorly defined.

**Objective:**

To identify the cell types that are regulated by the circulating microRNAs linked to asthma exacerbations and the extent to which the cells are regulated by miRNAs.

**Methods:**

We integrated a curated panel of exacerbation-associated circulating miRNAs with single-cell RNA sequencing (scRNA-seq) profiles from induced sputum of 16 asthma patients and 8 healthy controls. Experimentally validated miRNA-target interactions were combined with cell-type-specific differential expression. Elastic Net regression and SHAP analysis quantified gene-level regulatory contributions, yielding a composite Regulation Strength metric. Findings were validated against four independent GEO datasets.

**Results:**

Immune cells, including monocytes, dendritic cells, and macrophages, demonstrated the strongest statistically significant miRNA regulatory signals, in contrast to airway epithelial cells.hsa-miR-222-3p showed opposing regulatory effects in mature versus alveolar macrophages, indicating differentiation-state-dependent activity, while B_Plasma cells showed no detectable regulatory effect from any miRNA tested. Independent GEO validation confirmed higher expression of protective miRNAs (hsa-miR-126-3p, hsa-miR-146b-5p) in healthy individuals, consistent with prior CAMP cohort associations.

**Conclusion:**

Circulating miRNAs show cell-type-specific regulatory activity, strongest in monocytes, dendritic cells, and macrophages. hsa-miR-222-3p showed opposing regulatory directions between macrophage subtypes, while B_Plasma cells showed no effect, validated across independent GEO cohorts.

## Full Text Availability

The license terms selected by the author(s) for this preprint version do not permit archiving in PMC. The full text is available from the preprint server.

